# Quantitative proteomics reveals FLNC as a potential progression marker for the development of hepatocellular carcinoma

**DOI:** 10.18632/oncotarget.11921

**Published:** 2016-09-09

**Authors:** Yingzi Qi, Feng Xu, Lingsheng Chen, Yanchang Li, Zhongwei Xu, Yao Zhang, Wei Wei, Na Su, Tao Zhang, Fengxu Fan, Xing Wang, Xue Qin, Lingqiang Zhang, Yinkun Liu, Ping Xu

**Affiliations:** ^1^ State Key Laboratory of Proteomics, National Center for Protein Sciences Beijing, Beijing Proteome Research Center, Institute of Radiation Medicine, Beijing 102206, China; ^2^ Key Laboratory of Combinatorial Biosynthesis and Drug Discovery (Wuhan University), Ministry of Education and Wuhan University School of Pharmaceutical Sciences, Wuhan 430072, China; ^3^ State Key Laboratory for Conservation and Utilization of Subtropical Agro-Bioresources, Guangxi University, Nanning 530005, China; ^4^ Anhui Medical University, Hefei 230032, China; ^5^ Cancer Research Center, Institutes of Biomedical Sciences, Fudan University, Shanghai 200032, China; ^6^ Department of Clinical Laboratory, First Affiliated Hospital of Guangxi Medical University, Nanning 530021, China; ^7^ Institute of Microbiology, Chinese Academy of Science, Beijing 100101, China

**Keywords:** hepatocellular carcinoma (HCC), iTRAQ, filamin C (FLNC)

## Abstract

Hepatocellular carcinoma (HCC) caused by hepatitis B virus (HBV) infection is one of the most life-threatening human cancers in China. However, the pathogenesis of HCC development is still unclear. Here, we systemically analyzed liver tissues from different stages of HCC patients through 8-plex Isobaric Tags for Relative and Absolute Quantitation (iTRAQ) approach. A total of 4,620 proteins were identified and 3,781 proteins were quantified. When T1, T2 and T3 tumor tissues were compared with T1 non-tumor cells, 330, 365 and 387 differentially expressed proteins were identified respectively. IPA (Ingenuity Pathway Analysis) analysis revealed that these differentially expressed proteins were involved in endothelial cancer, cell spreading, cell adhesion and cell movement of tumor cell lines pathway and so on. Further study showed that the filamin C (FLNC) protein was significantly overexpressed with the development of HCC, which might play an important role in HCC invasion and metastasis. These results were also confirmed with western blot (WB). The mRNA levels were significantly increased in 50 pairs of tumor and adjacent non-tumor tissues from TCGA database. The higher expression of FLNC in HCC might be a common phenomenon, thereby shedding new light on molecular mechanism and biomarker for the diagnosis purpose of HCC development.

## INTRODUCTION

Hepatocellular carcinoma (HCC) is the fifth-most frequently diagnosed cancer in men, also the second-leading cause of cancer mortality worldwide [1]. It's also one of the most life threating diseases. Countries in the Asia-Pacific region and China tend to have the highest prevalence of hepatitis B infection worldwide which is often due to chronic hepatitis B virus (HBV) infection [2].

To date, potentially curative treatments for HCC include hepatectomy, transplantation, or local ablative therapy [3]. While these treatments are promising, the clinical practice has shown that the patients treated for early HCC lesions can have high survival rates and low chances of recurrence and metastasis. Currently Alpha-fetoprotein (AFP) is one of the biomarkers used widely for early diagnosis of HCC. However, the lack of specificity and sensitivity of AFP as a diagnostic marker limits the universality of its application [4, 5]. In addition, the recurrence after resection and personalized treatment of HCC are still very challenging in clinical practice. Deep understanding of the mechanism behind the onset and development of HCC induced by HBV will help to solve these problems [6].

Protein is the executor for nearly all biological processes. In 2000, proteomics was introduced into the study of the molecular mechanisms of HCC [7]. Zhang *et al.* conducted the silver-stained 2D gel-based comparative proteomics to analyze the highly differentiated proteome of hepatitis B virus (HBV)-infected tumor and adjacent non-tumor tissues [8]. Among these differentially expressed proteins, Hsp27 was up-regulated in well-differentiated HBV-infected HCC and the high expression of Hsp27 may indicate high differentiation of HCC. However, the low throughput of this technology makes it difficult to present a panoramic view of status and trend of proteome profiles behind HCC onset and development. The number of tumor lesions is one of the important references for HCC staging since it highly relates to the degree of malignancy of the disease. In general, multiple tumor lesions are corresponding to late stage of tumor-node-metastasis (TNM) and poor prognosis. Xing *et al.* compared the overall proteome profiles between the primary HCC with single and multiple lesions using iTRAQ-based quantitative proteomics approach [9]. The results revealed that the dysregulated proteins in multiple lesions group (MC group) were concentrated in Ubiquitin C (UBC) and NFκB signaling pathway but the dysregulated proteins in single lesion group (SC group) were more concentrated in ERK and NFκB signaling pathway. These findings indicate that different molecular mechanisms may be involved in the tumorigenesis and development of HCC.

In this study, we conducted a high coverage quantitative proteomics study on the tissues of three distinct stages of tumor-node-metastasis (TNM) using the iTRAQ-based quantitative proteomics approach. In total, 4,620 proteins were identified. The quantitative results indicated the protein heterogeneity and the number of differentially expressed protein increased with the development of HCC. The further analysis showed that 132 proteins were up-regulated and 198 were down-regulated in T1 tumor tissue, 172 proteins up-regulated and 193 down-regulated in T2 tumor tissue, 185 proteins up-regulated and 202 down-regulated in T3 tumor tissues compared to T1 non-tumor tissues. These differentially expressed proteins were involved in metabolism of exogenous substrate pathway, metabolism of vitamin pathway, cell death pathway, hepatic steatosis, endothelial cancer, proliferation of tumor cell pathway, oxidative stress, cell spreading, cell adhesion and tumor metastasis pathway. Subsequent analysis showed that the filamin C (FLNC) protein was significantly up-regulated in the tumor tissue compared with the adjacent non-tumor tissues and the expression level increased gradually with the tumor development. The result has been validated by western blot analysis. This was also confirmed with the RNA-Seq data from 50 independent HCC tumor and adjacent non-tumor tissues in TCGA database.

## RESULTS

**Figure 1:**
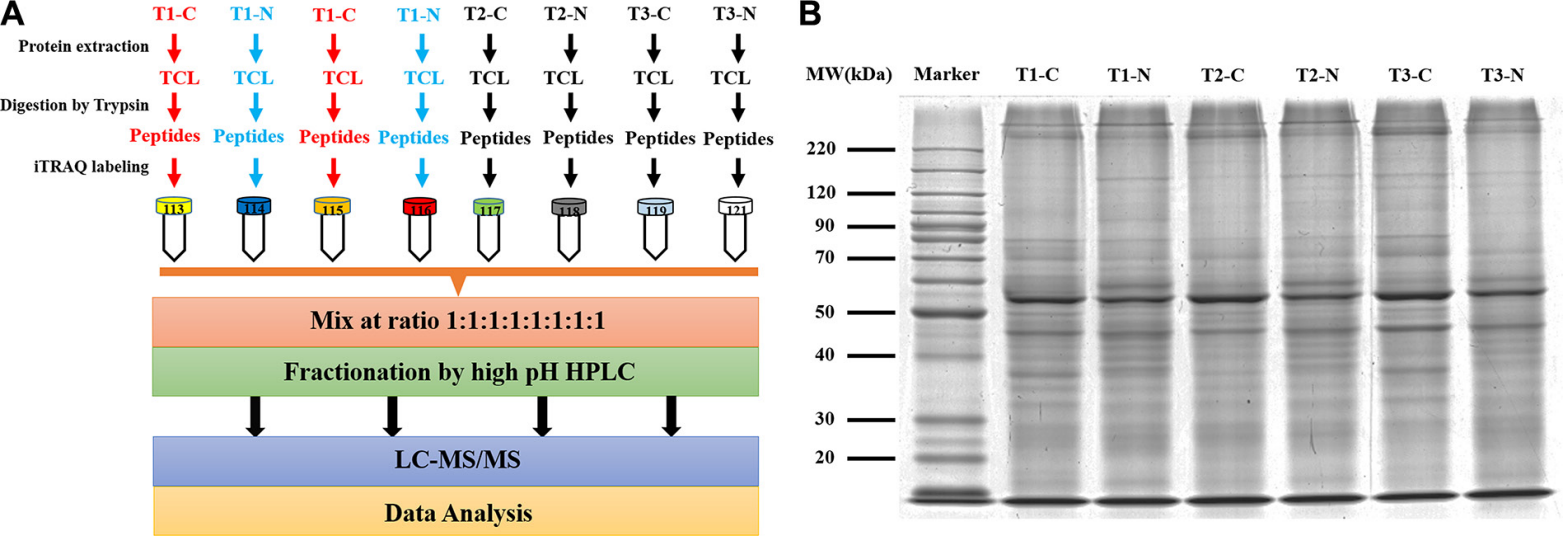
The workflow of quantitative proteomics study for a series of different stage HCC samples (**A**) The workflow of the quantitative proteomics analysis using 8- plex iTRAQ. T1-C and T1-N were used as technical replicates. (**B**) Separation of HCC protein by 10% SDS-PAGE gel.

To decipher the molecular mechanism underlying high malignance potential hepatocellular carcinoma, we designed a quantitative proteomics strategy to compare liver tumor tissue and pericarcinomatous tissue using the 8-labeled iTRAQ method. The experimental procedure was illustrated in Figure 1A. Sixteen paired samples including both tumor and adjacent non-tumor tissues were classified into three phases including T1N0M0, T2N0M0 and T3N0M0 according to the tumor-node-metastasis (TNM) classification of malignant tumors provided by the International Union Against Cancer (UICC). Table 1 shows the clinical indicators of each patient. After preparing total cell lysate (TCL) from individual sample, equal amount of protein from 5 to 6 patient livers of the same phase was pooled to reduce the individual variation [10]. The pooled samples were named as T1N0M0-Cancerous (T1-C), T1N0M0-Noncancerous (T1-N), T2N0M0-Cancerous (T2-C), T2N0M0- Noncancerous (T2-N), T3N0M0-Cancerous (T3-C), and T3N0M0-Noncancerous (T3-N), respectively (Figure 1A).

**Table 1:** Clinical characteristics of hepatocellular carcinoma patients

Symbol Number	Sex	Age	Tumor Size (cm)	TNM
1	Male	49	3	T1N0M0
2	Male	44	4	T1N0M0
3	Male	70	3.5	T1N0M0
4	Male	48	3.5	T1N0M0
5	Male	49	3.5	T1N0M0
6	Male	60	6	T1N0M0
7	Male	28	1.5	T2N0M0
8	Male	55	5	T2N0M0
9	Male	42	3.5	T2N0M0
10	Male	49	3	T2N0M0
11	Male	66	3	T2N0M0
12	Male	38	20	T3N0M0
13	Male	54	6	T3N0M0
14	Male	43	7	T3N0M0
15	Male	41	9.5	T3N0M0
16	Male	46	12	T3N0M0

### Quantitative proteomics analysis with a series of stepwise higher malignance potential hepatocellular carcinoma samples

As shown in Figure 1B, the six samples representing different stages of HCC showed similar staining intensity and pattern of bands, which indicated the high quality of the protein samples (Figure 1B). The samples were labeled with 113, 115, 117, 118, 119, and 121, respectively as indicated in Figure 1A. To determine the true biological relevance, both T1-C (red color) and T1-N (blue color) were split into two identical portions during in-gel digestion, followed by iTRAQ labeling with 114 and 116 respectively as technical replicates (Figure 1A). To facilitate the hydrophobic protein solubility, 8 M urea was used for protein extraction. Because high concentration of denaturant may inhibit trypsin activity and interfere with iTRAQ reagents, a short gel electrophoresis prior to digestion was introduced. After in-gel trypsin digestion and iTRAQ labeling, the labeled peptides were mixed and separated into 36 fractions by off-line high pH reverse phase (RP) chromatography (Figure 1A). Each fraction was analyzed by LC-MS for protein identification and quantification. The LC-MS analysis was repeated twice. Figure S1 showed the similar pattern and intensity between these repeats, which indicates LC-MS analysis was highly reproducible in our study.

In the iTRAQ experiment, 839,305 spectrums were acquired. Totally, we identified 33,503 peptides with MS identification success rate of 16.53% and 4,620 protein groups (Supplementary Table S1) with FDR on both of the peptides and proteins lower than 1%. GO analysis showed that the identified proteins were distributed in different cell components including the cytosol, envelope, subcellular organelle and extracellular space, suggesting the success of our extraction method. (Supplementary Table S2).

### Screening for the differentially expressed proteins

**Figure 2:**
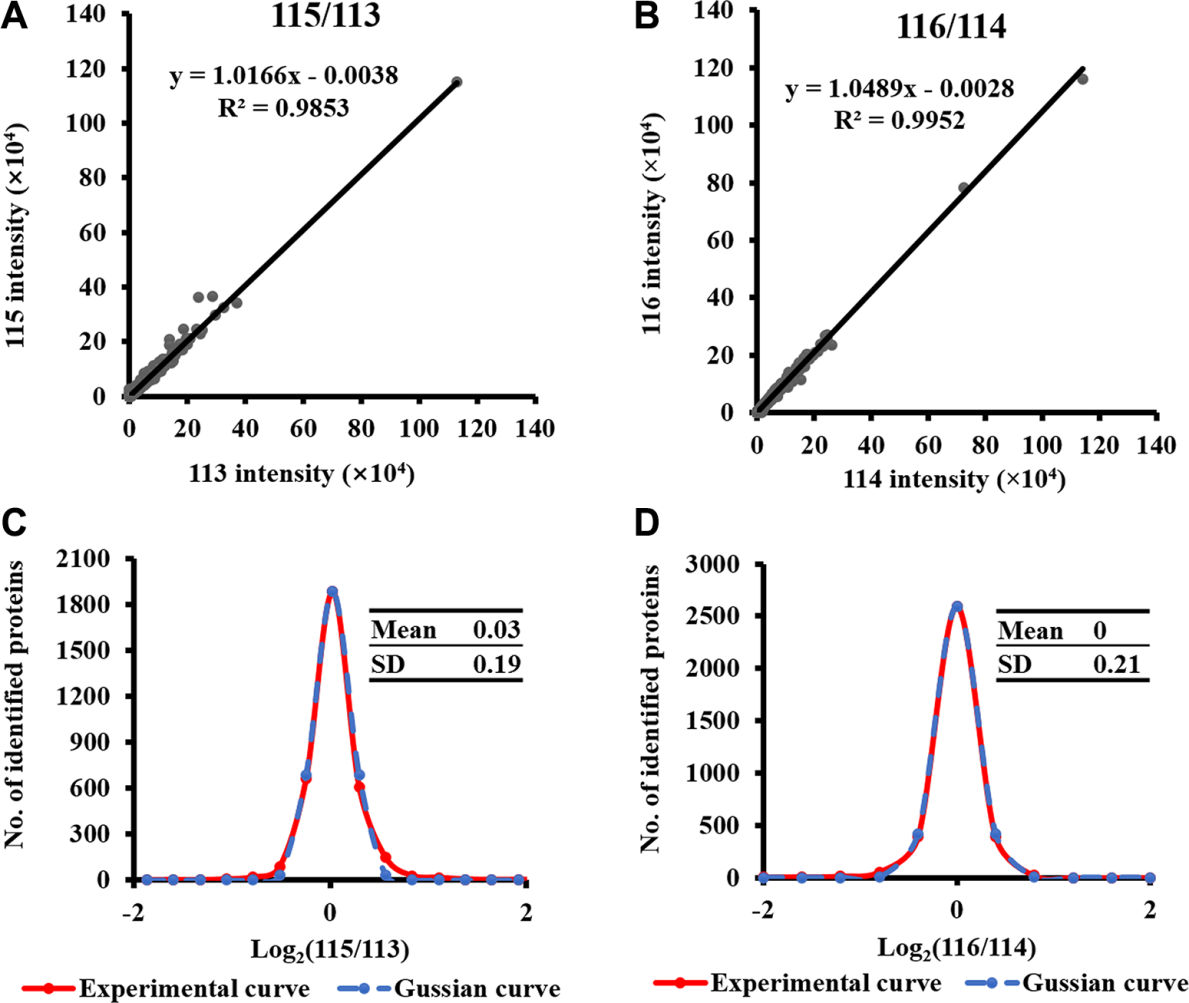
High correlation between the two technical replicates (**A**) The scatterplot of technical replicates labeled with 113 and 115 for TC-1. The Pearson correlation coefficient is 0.9784. (**B**) The scatterplot of technical replicates labeled with 114 and 116 for T1-N. The Pearson correlation coefficient is 0.9983. (**C**) The Gaussian fitting curve of log2 ratio of the intensities of 115/113. The red and blue curves represent the experimental and Gaussian fitting curve, respectively. (**D**) The Gaussian fitting curve of log_2_ ratio of the intensities of 116/114. The red and blue curves represent the experimental and Gaussian fitting curve, respectively.

In 4,620 identified proteins, 3,781 proteins were successfully quantified, in which 3,475 proteins were quantified with more than one peptide. A summary of the quantitative proteins was presented in Supplementary Table S1. To determine the measurement error, the same tumor and non-tumor tissues from T1 phase were analyzed twice as technical replicates, which were labeled as 113 and 115 for tumor T1 tissues and 114 and 116 for non-tumor T1 tissues, respectively. The results showed high correlation between the paired technical replicates. The R-square of the regression between 113 and 115 was 0.9784 and the slope was 1.0161 (Figure 2A–2B). The R-square of the regression between 114 and 116 was 0.9937 and the slope was 1.0644. Both log_2_ ratios of 113 vs 115 and 114 vs 116 follow Gaussian distribution with N (0.025, 0.036) and N (0, 0.0441) respectively. These results implied a high reproducibility and a well-controlled experimental process during our proteomics study.

**Figure 3:**
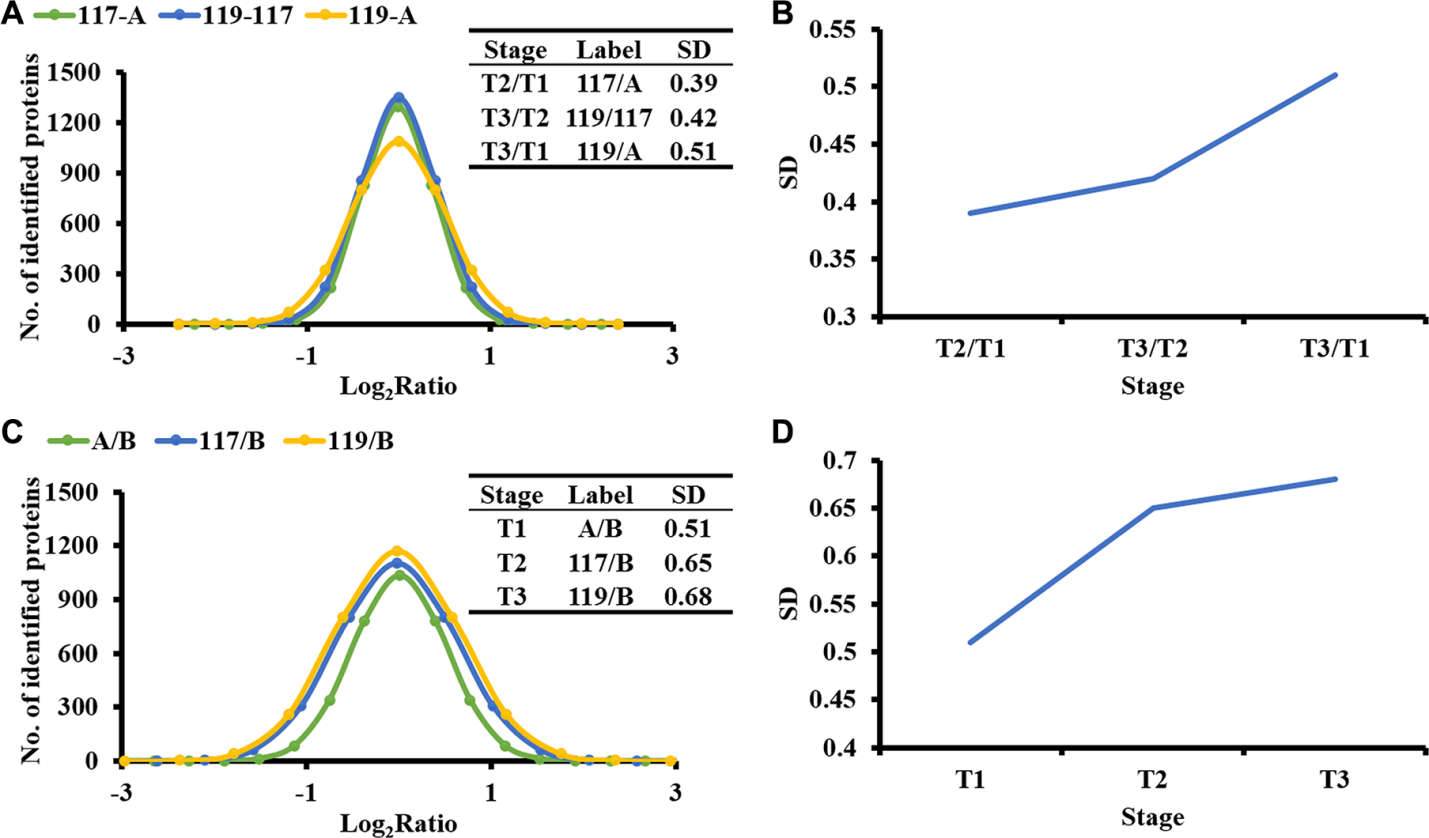
The heterogeneity of proteome samples increase with tumor development (**A**) The Gaussian fitting curves of the log_2_ intensity ratios of T2-C/T1-C, T3-C/T2-C, and T3-C/T1-C. (**B**) The SD (standard deviation) of log_2_ intensity ratios for the tissues of T2-C/T1-C, T3-C/T2-C, and T3-C/T1-C. (**C**) The Gaussian fitting curves of log_2_ intensity ratios for the tissues of T1-C/T1-N, T2-C/T1-N, and T3-C/T1-N. (**D**) The SD of log_2_ intensity ratios for the tissues of T1-C/T1-N, T2-C/T1-N, and T3-C/T1-N.

The log_2_ ratios of the quantified proteins in tumor tissue T2 vs T1, T3 vs T2 and T3 vs T1 were calculated and fitted to Gaussian distributions. The standard deviations (SDs) of the 3 distributions were 0.39, 0.42 and 0.51, respectively, which showed a trend of increasing with tumor development (Figure 3A–3B). The log_2_ ratios of the quantified proteins in tumor tissue T1, T2 and T3 vs T1 non-tumor tissue were fitted to Gaussian distributions. The SDs of the distribution were 0.51, 0.65 and 0.68 respectively, which increased as well. The rising trend of SDs in these tissues indicates the proteome heterogeneity increases with tumor development.

**Figure 4:**
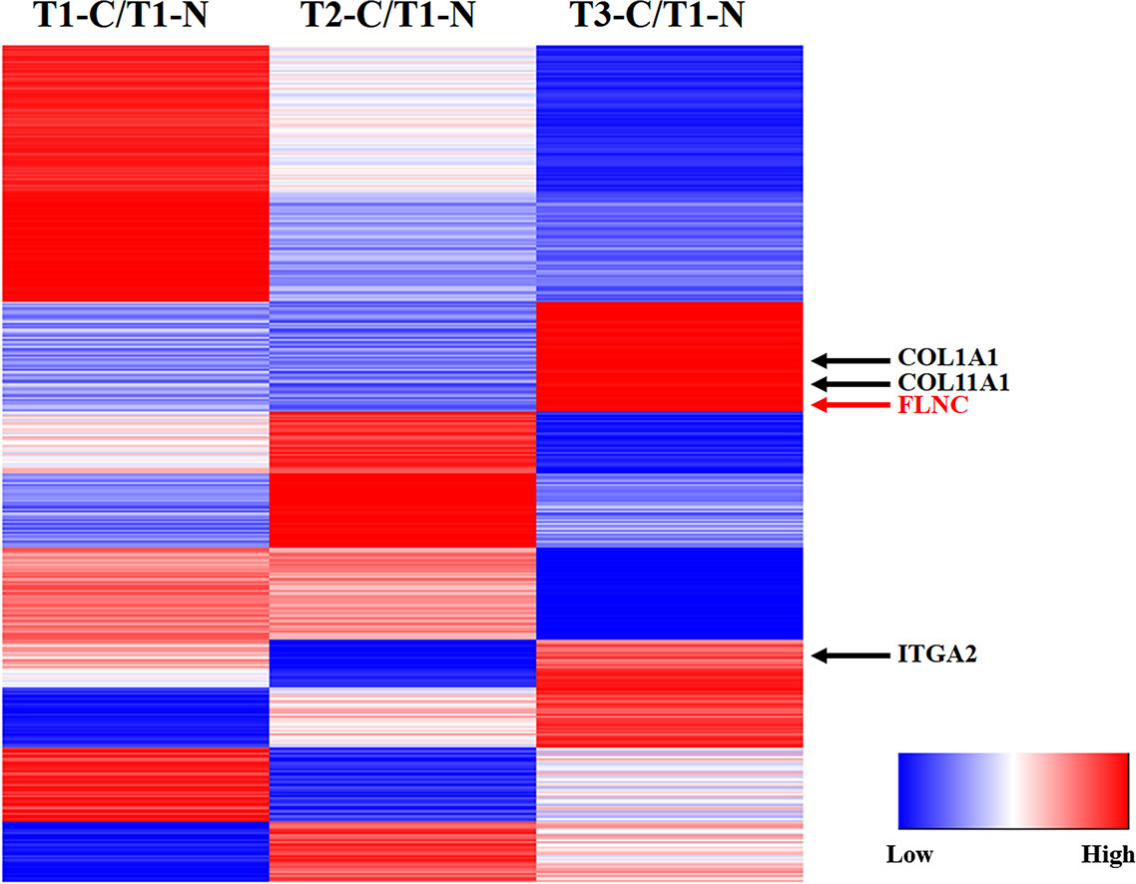
K-means cluster of differentially expressed proteins (detailed information were shown in Table S6).

By setting two fold changes as threshold for significant up- or down-regulation of proteins, we identified 132 up-regulated and 198 down-regulated proteins in T1 tumor tissue, 172 up-regulated and 193 down-regulated proteins in T2 tumor tissue, 185 up-regulated and 202 down-regulated proteins in T3 tumor tissues compared to T1 non-tumor tissues respectively (Table 2). There were 120 common proteins among these three samples (Figure S2A). The detailed information of differentially expressed proteins was shown in Supplementary Table S3–S5, and its expression were clustered in heat map based on K-means algorithm (Figure 4 and Supplementary Table S6).

**Table 2:** List of differentially expressed proteins between different groups

Sample	Differentially Expressed Proteins	Up-Regulated Proteins	Down-Regulated Proteins
T1-C/T1-N	330	132	198
T2-C/T1-N	365	172	193
T3-C/T1-N	387	185	202

### The relevance of differentially expressed proteins with the stepwise increased malignance of HCC

To explore the biological function of the differentially expressed proteins, the IPA analysis was performed. The up-regulated proteins in T1 tumor compared to T1 non-tumor tissues were involved in the endothelial cancer, mass liver, hepatomegaly, oxidative stress, tumor and cell proliferation; the down-regulated proteins were related to immune response (Figure S2B). The up-regulated proteins in T2 tumor compared to T1 non-tumor tissues were involved in immune cells recruitment, and RNA virus infection. The down-regulated proteins were involved in external toxicity metabolism, lipid metabolism, and small molecule metabolism (Figure S2C). The up-regulated proteins in T3 tumor compared to T1 non-tumor tissues were involved in cells movement, and the down-regulated proteins were involved in carbohydrates metabolism, external toxicity metabolism, lipid metabolism, small molecule metabolism, cell death, lipid metabolism, and energy metabolism (Figure S2D).

**Figure 5:**
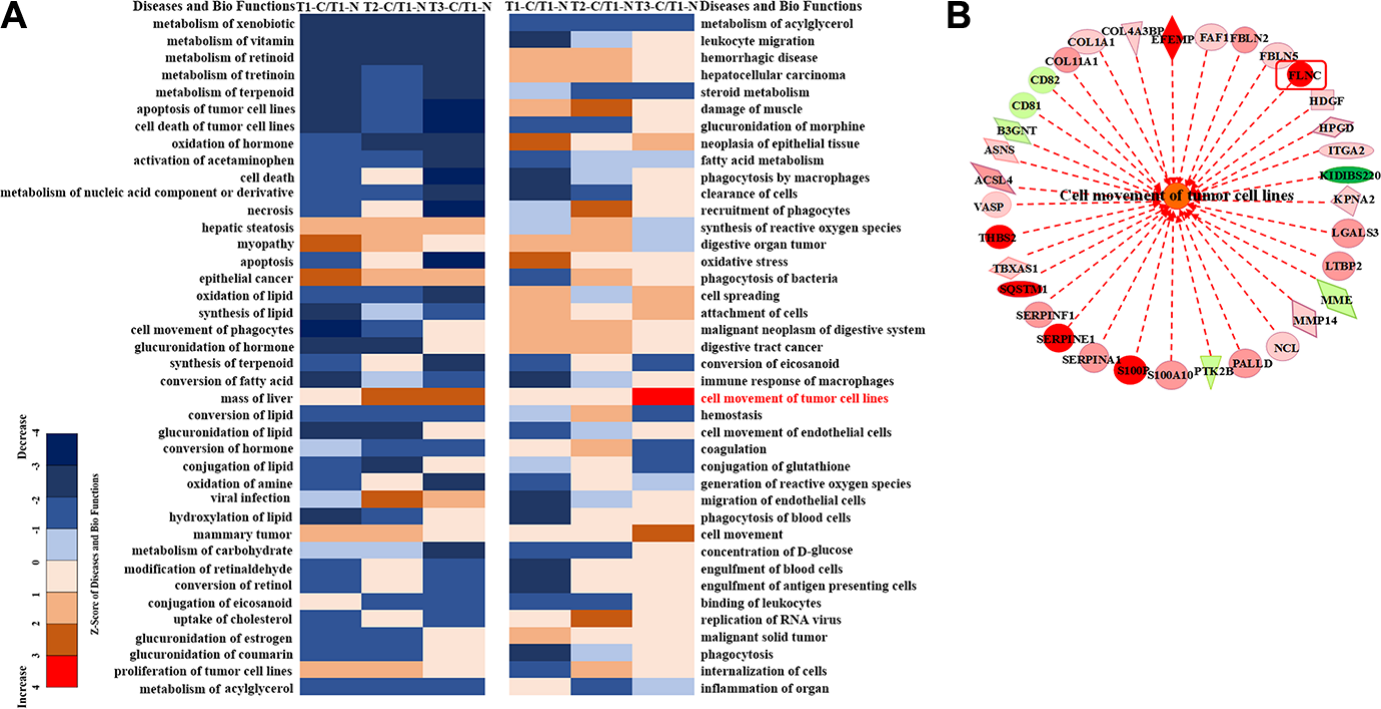
The result of IPA analysis revealed that FLNC might play important role in the tumor development (**A**) The comparative analysis of diseases and function on T1-C/T1-N, T2-C/T1-N and T3-C/T1-N through IPA. (**B**) Schematic diagram of the genes involved in the cancer cell migration pathway.

The profiles of differential protein expressions in T1, T2 and T3 tumor vs T1 non-tumor tissue using IPA analysis were compared. The results showed that exogenous substrate pathway, metabolism of vitamin pathway, cell death pathway significantly down-regulated in T1, T2 and T3 tumor cells compared with T1 non-tumor tissue. However, the cancer related pathways such as hepatic steatosis, endothelial cancer, proliferation of tumor cell pathway, oxidative stress, cell spreading, cell adhesion and tumor metastasis pathway were up-regulated in T1, T2 and T3 tumor tissues compared with T1 non-tumor tissue. Especially tumor cell metastasis pathway was the largest increase in the T3 tumor tissue (Figure 5A). We have studied series of proteins involved in this pathway. One of the proteins, FLNC, has been reported to be relevant to cancer (Figure 5B).

FLNC is a member of the filamin protein family and exists as dimer in muscle-specific cells. Functioning as a large actin-cross-linking protein, it is involved in cell contraction and spreading [11]. There were several studies try to reveal the relevance of FLNC with cancer. Jie Qiao *et al.* found that FLNC is down-regulated in gastric cancer cell lines compared with normal gastric cells [12]. Masayo Adachi-Hayama's study showed that FLNC expression was up-regulated in the glioma tissue compared with normal brain tissues and its expression level was positively correlated with the tumor histological grade [13]. However, the FLNC expression level in HCC and its roles in tumor cell invasion and metastasis are unclear.

### Validation of FLNC' expression pattern and function

**Figure 6:**
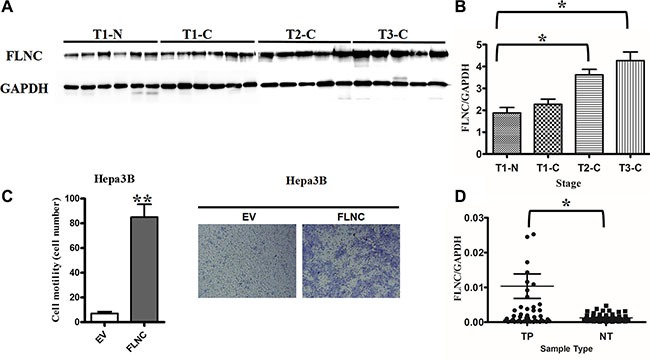
Validation of FLNC's expression pattern and function in cell migration (**A**) The western blot for FLNC protein expression in T1-N (6 samples), T1-C (6 samples), T2-C (5 samples), and T3-C (5 samples). (**B**) Quantitative histogram of the western blot results. (C) The transwell cell migration assay to investigate the role of FLNC in Hepa3B migration. Representative images were shown in left panel and migratory cells were counted in 3 non-overlapping frames of the membrane (right panel). (**D**) The scatterplot of mRNA expression of FLNC in the 50 pairs of tumor and non-tumor tissues collected from TCGA RNA-Seq database.

The FLNC expression in the tissue samples was validated using western blot. To investigate the FLNC protein level in each individual, 6 T1 tumor, 6 T1 non-tumor, 5 T2 tumor and 5 T3 tumor tissues were chosen for western blot study. The intensity of GAPDH was similar in all of these tested samples, indicating the similar amount of sample has been loaded onto the gel (Figure 6A). However, the average FLNC expression in T1 non-tumor, T1, T2 and T3 tumor tissues increased over 50% (Figure 6B). The results suggest that the expression level of FLNC protein may exhibit a positive correlation with the invasion and metastasis of HCC. *Filamin* C gene was ectopically expressed in Hepa3B cells and cell migration ability was analyzed in a Transwell plate. Ectopic expression of FLNC significantly enhanced the migration ability of Hepa3B cells (Figure 6C).In order to understand if this conclusion has broader implications, we chose the RNA-Seq of fifty pairs of tumor and adjacent non-tumor tissues from TCGA database to investigate the FLNC mRNA expression using GAPDH as internal control. The results revealed that FLNC mRNA expression in tumor tissues was significantly higher than that in adjacent non-tumor tissues (Figure 6D).To further explore the dynamic expression of tumor invasion and metastasis related proteins, we reanalyzed the KEGG pathway of differential proteins in T3 tumor tissue vs T1 non-tumor tissue group. Results showed that these proteins were significantly enriched in antigen processing and presentation, ECM-receptor interaction, and focal adhesion pathway. ECM-receptor interaction and focal adhesion pathways were closely related to tumor invasion and metastasis, in which three proteins of ITGA2, COL1A1 and COL11A1 were also behaved up-regulation trend in cancer, except FLNC. We compared the RNA-seq datasets of 50 liver cancer patients obtained from TCGA database. Likewise, we found that the expression levels of ITGA2, COL1A1 and COL11A1 in tumor tissues were obviously higher than non-tumor tissues (Figure S3B–S3D), which illustrates that tumor invasion and metastasis related pathways were up-regulated in tumor compared with non-tumor tissues, especially for FLNC.These results further demonstrated that the up-regulation of FLNC is a common phenomenon in HCC and FLNC might have the potential to be used as a biomarker for HCC diagnosis.

## DISCUSSION

In this study, the iTRAQ-based quantitative proteomics approach was used to study the tumor tissues and the adjacent non-tumor tissues at different developmental stages of HCC. In total, 4,620 proteins were identified and 3,781 proteins were quantified, in which there were 3,475 proteins with more than two peptides. IPA software was used to conduct the bioinformatics analysis of the differentially expressed proteins in T1, T2 and T3 tumor tissues compared with T1 non-tumor tissue. The results revealed that the proteins involved in the pathways related to metabolism of exogenous substrate, metabolism of vitamin and cell apoptosis were down-regulated in T1, T2 and T3 tumor tissues compared to T1 non-tumor tissues. However, the proteins involved in the cancer related pathways including hepatic steatosis, endothelial cancer, proliferation of tumor cell, oxidative stress, cell spreading, and cell adhesion were up-regulated in all three tumor tissues compared to T1 non-tumor tissues. It's noteworthy that the tumor metastasis pathway showed the largest increase of protein expression in T3 tumor tissue. After analyzing the proteins involved in this pathway, it was found that FLNC protein might be the target molecule for HCC invasion and metastasis.

The challenges in HCC include late diagnosis, high risk of recurrence and metastasis and poor prognosis. Therefore, early prevention, diagnosis, intervention and treatment have significant impacts on the tumor development, treatment effectiveness, and prognosis and survival time [14]. All these highlight the need to identify valuable biomarkers for the diagnosis and treatment of HCC. Alpha-fetoprotein (AFP) is one of the most important biomarkers, which have been used widely for early diagnosis of HCC. AFP combined with imaging diagnosis has become the most powerful tools for HCC diagnosis and detection. However, 30–40% of HCC patients showed AFP-negative which might be due to HCC heterogeneity. Thus, the lack of specificity and sensitivity of AFP as a diagnostic marker create the urgency to develop new biomarkers for HCC early diagnosis. The present study revealed the possibility to use FLNC as potential biomarkers during the development of HCC.

FLNC, as a member of the filamin protein family, exists as dimer in muscle-specific cells. Functioning as a large actin-cross-linking protein, it is involved in cell contraction and spreading. Two other proteins, filamin A (FLNA) and filamin B (FLNB), are also parts of the filamin family. They interact with actin as cytoskeleton regulator and are involved in many cellular processes including cancer cell invasion and metastasis. The filamin proteins are also known to serve as scaffolds for many binding partners including channels, receptors, intracellular signaling molecules, and even transcription factors [15]. It has been found that the C terminal of FLNA can serve as docking sites for intracellular signaling molecules such as RalA, Rac, Rho and CDC42 as well as many transmembrane proteins such as sarcoglycan (SGCD), presenilin (PSEN), caveolin-1(CAV1), integrin β1(ITGB1) and so on [16]. They integrate both cellular architectural and signaling functions and are essential for cancer development which includes cancer cell migration and DNA damage [17].

Previous studies have shown that filamin family is closely related to multiple type of tumorigenesis and carcinoma development [11]. Interestingly, the role of FLNC, another member in the filament protein family, is not clear in tumor cell invasion and metastasis. Jie Qiao *et al.* found that FLNC was down-regulated in gastric cancer cell lines compared with normal gastric cells [12]. Atsushi Kaneda's study suggested that the methylation of CpG island in the 5′ terminal of FLNC may play an important role in the downregulation of FLNC in the cancer cell [18]. Takeshi Nakajima *et al.* further indicated the increase of FLNC methylation level may be due to Helicobacter pylori (HP) infection [19]. Masayo Adachi-Hayama's study showed that FLNC expression was up-regulated in the glioma tissue compared with normal brain tissues and its expression level was positively correlated with the tumor histological grade [13]. Anti-FLNC antibody was also detected in the serum of glioma patients, but its levels were inversely correlated with the tissue expression. FLNC can only be detected around the nucleus in normal glial cells, whereas it spreads into the entire cytoplasm in tumor cells. These indicate the subcellular localization of FLNC might play an important role for its function although it does not enter into the nucleus as the other members in filament protein family does.

Invasion and metastasis of tumor cells is a complex process including the dissociation of the cell from the primary tumor, degradation of extracellular matrix, migration, intravasation, movement and survival in the blood circulatory system, extravasation and proliferation at the new location. Functioning as actin-cross-linking protein, FLNC serves as a cytoskeleton protein to play a key role in providing mechanical strength and supporting cell morphology changes which can facilitate the metastasis of tumor cells. Besides FLNC, ITGA2, COL1A1 and COL11A1 were tumor invasion and metastasis-related proteins. TCGA tumor and non-tumor tissue comparative analysis (50 samples) showed that these three proteins behaved higher expression in tumor tissue. COL1A1 and COL11A1 are collagens, COL1A1 belongs to type I and COL11A1 belong to type XI. Collagens is one kind of extracellular matrix, which acts as the frame structure playing a supportive role and protecting the integrity of tissues and organs [20]. Most members of collagens are closely associated with the genesis and the development of several malignancies, so COL1A1 behaves high expression in prostate, esophageal, brain tumor, pancreatic many malignant tumors [21–23]. In addition, type I collagen might have a tight relation to liver fibrosis [24]. There has been a relatively large current controversy on the COL11A1 expression in the development of tumors [25–27]. In our datasets, results showed that CLL11A1 were significantly up-regulated in T3 tumor tissues, while it didn't behave the similar high up-regulation in TCGA RNA-seq data of tumor and non-tumor samples. Therefore, the expression of CLL11A1 in the tumor development might be affected by clinical case, disease causation and patterns, and so on. ITGA2 is a membrane of the integrin-binding family, which is also a significant adhesion molecule in tumor cell invasion [28]. The expression of integrin-binding and collagen proteins finish invasion and metastasis after ECM invasion of tumor. In cell, the expression of FLNC might be controlled by the upstream regulatory protein. FLNC might promote the invasion and metastasis of tumor cells by regulating the expression of integrin-binding and collagen proteins. The impact of FLNC on invasion and metastasis remains unclear and needs further investigation. As a scaffold protein, FLNC needs to interact with other proteins to achieve its function. Therefore, the understanding of FLNC binding proteins and signaling pathways will be crucial to elucidate the function of FLNC.

## MATERIALS AND METHODS

### Clinical samples

Liver tissues provided by Zhongshan Hospital (Shanghai, China) were obtained from 16 male patients who underwent a curative hepatectomy. All participants gave written informed consent for their participation. Tumor staging was determined according to the tumor-node-metastasis (TNM) classification of the International Union Against Cancer (UICC) by senior pathologists. Among these samples, 6 tissues were classified as T1N0M0, 5 tissues as T2N0M0, and 5 tissues as T3N0M0. Paired tissues were obtained from each patient, one from the adjacent non-tumor region (normal) and the other from the tumor region of the resected liver. Tissues were washed with PBS three times and immediately frozen in liquid nitrogen and stored at −80°C until use. The clinicopathological features of 16 HCC patients were shown in Table 1. Informed consent was obtained from all of the patients.

### Protein extraction for proteomic study

In each stage, we pooled liver tissues (50 mg) from 5 or 6 patients together, which were served as one sample for comparison. The liver tissue homogenates were lysed with the same lysis buffer containing 8 M Urea, 5 mM IAA, 50 mM NH_4_HCO_3_ and protein inhibitor cocktail followed by sonication (2s on, 4s off, 10min; 26.6 kHz, 30 W) and centrifugation (13,300 rpm, 10 min). The supernatants were collected and the protein concentration in the TCL was determined by both of Bradford assay and short SDS-PAGE stained with Coomassie Blue G-250.

### Protein gel-assisted digestion

The equal amount of lysates from each of pooled tissue samples (100 μg) were used. Before loading, the proteins were reduced with 10 mM DTT at 45°C for 30 min and alkylated with 20 mM iodoacetamide (IAA) in the dark at room temperature for 30 min. The proteins were run on a short 10% SDS-PAGE gel for approximately 0.8 cm. The gel was stained with Coomassie Blue G-250 and cut into approximately 1 mm^3^ cubes followed by distaining with 50% ACN in 25 mM NH_4_HCO_3_ and dehydrating with 100% ACN. In-gel digestion was performed using 10 ng/μL trypsin (Promega, Madion, WI) at 37°C overnight. After digestion, the peptides were extracted from the gel using sequential extraction with 100 μL of 5% (v/v) formic acid (FA) in 50% ACN once and 100 μL of ACN three times.

### iTRAQ labeling

The peptides were labeled with 8-plex iTRAQ Multiplex Kit according to the manufacturer's instructions (AB Sciex, Foster City, CA, USA). Briefly, the peptides were dissolved in 100 mM TEAB pH 8.5 solution before the labeling reagent was added. After a 2 hours' incubation, the reaction was quenched by adding an equal volume of water. Differentially labeled peptides were mixed and dried with a speed-vac.

### 2D LC-MS/MS

The first dimension high pH RP separation was performed as described previously [29]. Briefly, the solvent gradient was set as follows: 3% Buffer B, 5min; 3–5% B, 5 min; 5–18% B, 35min; 18–34% B, 22 min; 34–95% B, 11 min; 95–3% B, 2 min. The labeled tryptic peptides were separated at an eluent flow rate of 0.7 mL/min, and then the chromatogram was monitored at 214 nm. The column oven was set at 45°C. The eluent was collected every 1.5 min. In total, 53 fractions were collected and combined into 36 fractions. Finally, each fraction was lyophilized and reconstituted in 15 μL of 0.1% (v/v) FA, 1% (v/v) ACN in water for subsequent analysis.

The second dimension low pH RP chromatography was coupled with tandem mass spectrometry (LC-MS/MS) on an LTQ Qrbitrap Velos Mass Spectrometer (Thermo Fisher Scientific, Waltham, MA, USA) equipped with a nanoAcquity Ultra Performance LC (UPLC) system (Waters Corporation, Milford, MA, USA) with an in-house packed capillary column (75 μm i.d. ×15 cm) with 3 μm C_18_ reverse-phase fused-silica (Michrom Bioresources, Inc., Auburn, CA). The samples were loaded onto the column by an autosampler and eluted with a 100 min gradient covering 0–35% of Buffer B (Buffer A: 0.1% formic acid (FA) and 2% acetonitrile (ACN) in water; Buffer B: 0.1% FA in ACN) as described previously. The eluted peptides were ionized under high voltage (2.0 kV) and detected by the Orbitrap Velos mass spectrometer in a survey scan (400–1,800 m/z; 1 × 10^6^ automatic gain control (AGC) target; 150 ms maximum ion time, resolution 30,000 at m/z 400) followed by 10 data dependent scans fragmented via Higher Energy Collision Induced Dissociation (HCD) (2 m/z isolation width, 40% collision energy, 30,000 AGC target, 150 ms maximum ion time, dynamic range of 35 seconds).

### Data processing and database searching

All the raw data were processed using the MaxQuant software suite (version 1.5.1.2, Martinsried, Germany) against the Uniprot human reference protein database (version July, 2014). The target-decoy based strategy was used to control the peptide and protein false discovery rate (FDR). The search parameters were the following. Up to two missed cleavages were allowed for protease digestion and peptides with seven to twenty amino acids were retained. Precursor ion peaks were searched with an initial mass tolerance of 20 ppm and fragmented ion peaks were searched with an initial mass tolerance of 0.1 Da. Fixed modifications of Carbamidomethylation (C), iTRAQ 8-plex (K) and iTRAQ 8-plex (N-term) were specified. Oxidation of methionine was included as variable modification. The FDR was set to 0.01 for both of peptide and protein identifications.

### Bioinformatics analysis and TCGA analysis

Gene Ontology (GO) annotation and pathway analysis were performed using DAVID [30]. Ingenuity Pathway Analysis software (demo version 14400082, Ingenuity Systems, http://www.ingenuity.com) was used to assign the interaction network of the dysregulated proteins.

The mRNA expression data of FLNC was obtained from the Cancer Genome Atlas (TCGA) portal (http: //cancergenome. nih. gov/, last accessed on 04062015). We collect expression data of 50 HCC and adjacent normal tissues.

### Western blot analysis

Approximately 75 μg of protein lysate was separated on SDS-PAGE gels and transferred onto nitrocellulose membranes (Bio-Rad, Hercules, CA, USA). After blocking, the membranes were incubated with primary antibody and detected using a Tanon 5200 Multi imager (Tanon, Shanghai, China). Rabbit polyclonal Filamin C antibody was purchased from Sigma-Aldrich Inc (St. Louis, MO, USA), and GAPDH antibody was bought from Kangwei Century Co. LTD (Beijing, China). Protein up-regulation or down-regulation was determined by comparing the relative band density between the tumor and the paired normal tissues from the same patient (ScionImage software, Scion Corporation, http: //scion-image.software. informer.com/).

### Migration assay

The plasmid pcDNA3.1-FLNC was a gift from Professor Feng Liu [12]. For the transwell migration assay, after two days of transfection with pcDNA3.1-FLNC and empty vector, Hepa3B cells were resuspended in serum-free medium and seeded into the insert well of a 24-well plate (8-mm pores, BD biosciences, Beijing, China) for 24 h. The culture medium containing 10% fetal bovine serum was used as a chemoattractant and placed in the bottom chamber. Cells were fixed in paraformaldehyde (4%) and stained in crystal violet (0.5% in 20% methanol). Remaining cells in the upper chamber (non-migratory cells) were washed with a cotton swab and the membrane was removed. Adherent cells to the bottom of the membrane (migratory cells) were counted under an Olympus microscope (Olympus USA).

### Statistical analysis

Statistical analysis was performed using Prism software (v5.01, GraphPad software Inc.) and SPSS V17.0 (IBM Corporation, Armonk, NY). A Student's *t*-test was used to analyze the data for the quantitative Western blot assays, and the Wilcoxon Rank-Sum test was used to analyze the data for the TCGA database. *P* values less than 0.05 were considered significant.

## SUPPLEMENTARY MATERIALS FIGURES AND TABLES














