# Supplementary material for: Quantitative proteomics reveals FLNC as a potential progression marker for the development of hepatocellular carcinoma
Source: Oncotarget. 2016 Sep 9;7(42):68242–52. doi: 10.18632/oncotarget.11921 (PMC5354476; doi:10.18632/oncotarget.11921)
Supplement: Supplementary file 1 [file oncotarget-07-68242-s001.pdf]

Quantitative proteomics reveals FLNC as a potential progression marker for the development of hepatocellular carcinoma

Supplementary Materials

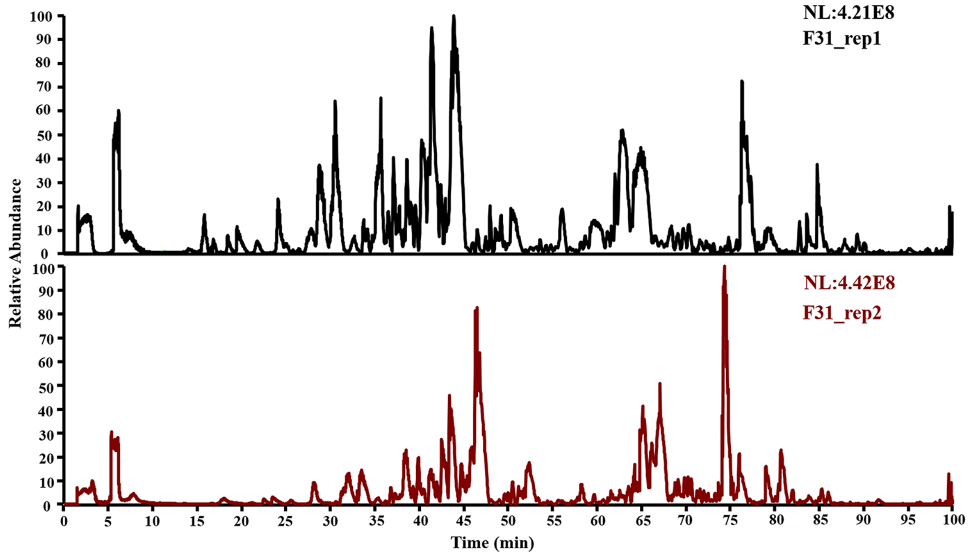

Supplementary Figure S1: Representative extracted ion current (XIC) analysis for LC-MS/MS on the iTRAQ labeled samples.

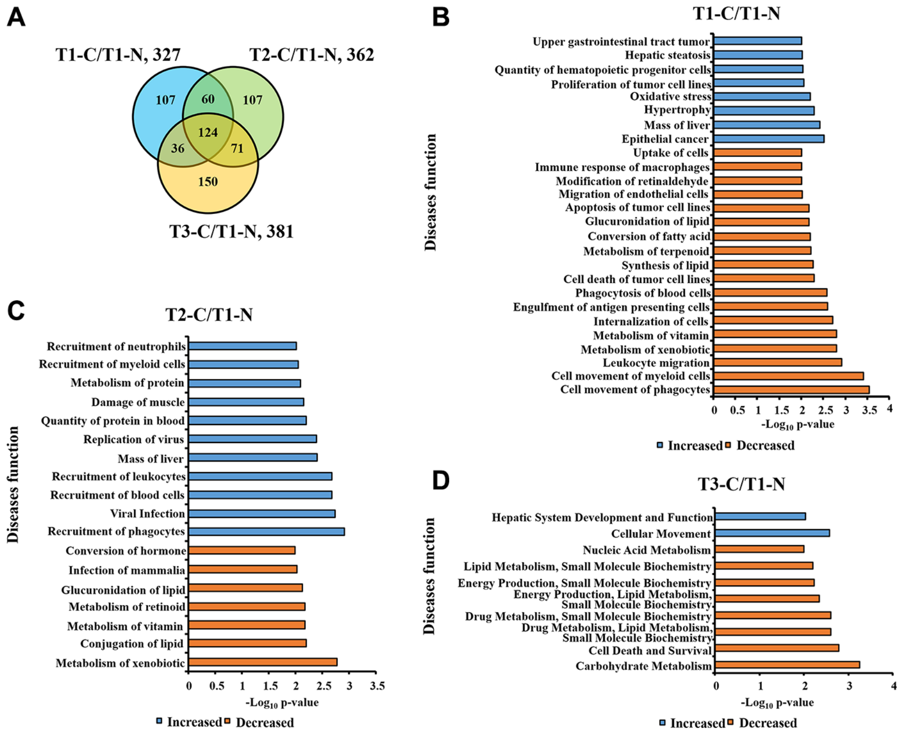

Supplementary Figure S2: Differential expression analysis for pathways. (**A**) The Venn diagram of the differentially expressed proteins in T1-C/T1-N, T2-C/T1-N, and T3-C/T1-N. (**B–D**) The diseases and function pathway analysis of the differentially expressed proteins in T1-C/T1-N, T2-C/T1-N and T3-C/T1-N, respectively.

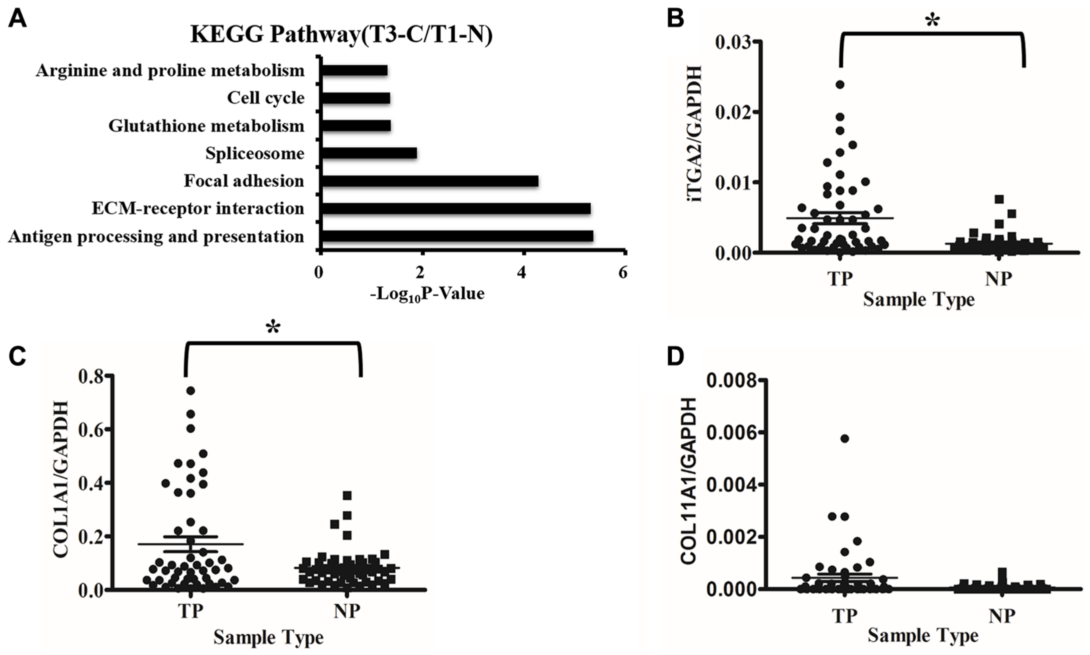

Supplementary Figure S3: iTGA2, COL1A1 and COL11A1 proteins were up-regulated in live cancer tissues, which belong to tumor invasion and metastasis-related proteins. (**A**) KEGG pathway analysis of T3-C vs T1-N differential proteins. (**B**) The scatterplot of mRNA expression of iTGA2 in the 50 pairs of tumor and non-tumor tissues collected from TCGA RNA-Seq database. (**C**) The scatterplot of mRNA expression of COL1A1 in the 50 pairs of tumor and non-tumor tissues collected from TCGA RNA-Seq database. (**D**) The scatterplot of mRNA expression of COL11A1 in the 50 pairs of tumor and non-tumor tissues collected from TCGA RNA-Seq database.

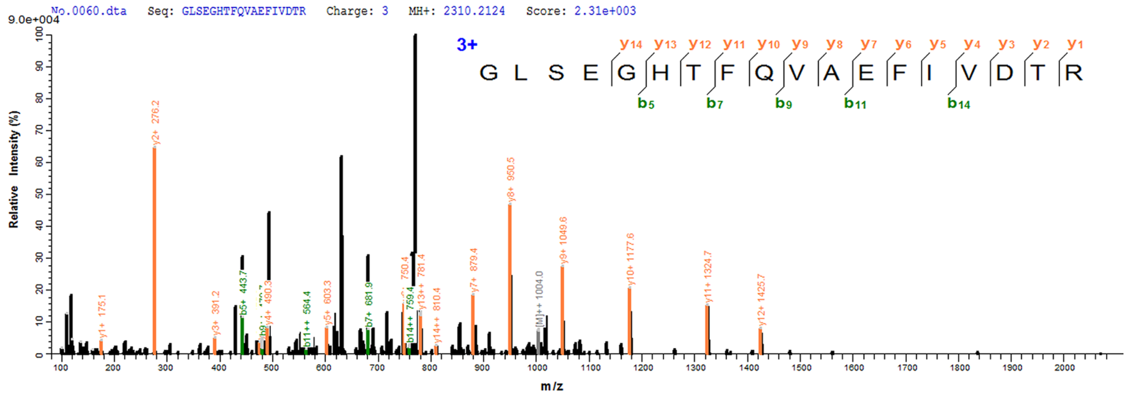

Supplementary Figure S4: Representative mass spectra of FLNC.

Supplementary Table S1: The list of quantitative proteins. See Supplementary_Table_S1

Supplementary Table S2: GO analysis for cell components of the identified proteins.
See Supplementary_Table_S2

Supplementary Table S3: The list of differentially expressed proteins between T1-C and T1-N.
See Supplementary_Table_S3

Supplementary Table S4: The list of differentially expressed proteins between T2-C and T1-N.
See Supplementary_Table_S4

Supplementary Table S5: The list of differentially expressed proteins between T3-C and T1-N.
See Supplementary_Table_S5

Supplementary Table S6: The K-Means clustering of differentially expressed proteins.
See Supplementary_Table_S6
